# Hox and ParaHox gene expression in early body plan patterning of polyplacophoran mollusks

**DOI:** 10.1002/jez.b.22671

**Published:** 2016-04-21

**Authors:** Martin Fritsch, Tim Wollesen, Andreas Wanninger

**Affiliations:** ^1^ Department of Integrative ZoologyFaculty of Life SciencesUniversity of ViennaVienna1090Austria

## Abstract

Molecular developmental studies of various bilaterians have shown that the identity of the anteroposterior body axis is controlled by Hox and ParaHox genes. Detailed Hox and ParaHox gene expression data are available for conchiferan mollusks, such as gastropods (snails and slugs) and cephalopods (squids and octopuses), whereas information on the putative conchiferan sister group, Aculifera, is still scarce (but see Fritsch et al., 2015 on Hox gene expression in the polyplacophoran *Acanthochitona crinita*). In contrast to gastropods and cephalopods, the Hox genes in polyplacophorans are expressed in an anteroposterior sequence similar to the condition in annelids and other bilaterians. Here, we present the expression patterns of the Hox genes *Lox5, Lox4*, and *Lox2*, together with the ParaHox gene *caudal* (*Cdx*) in the polyplacophoran *A. crinita*. To localize Hox and ParaHox gene transcription products, we also investigated the expression patterns of the genes *FMRF* and *Elav*, and the development of the nervous system. Similar to the other Hox genes, all three Acr‐Lox genes are expressed in an anteroposterior sequence. Transcripts of *Acr‐Cdx* are seemingly present in the forming hindgut at the posterior end. The expression patterns of both the central class Acr‐Lox genes and the *Acr‐Cdx* gene are strikingly similar to those in annelids and nemerteans. In Polyplacophora, the expression patterns of the Hox and ParaHox genes seem to be evolutionarily highly conserved, while in conchiferan mollusks these genes are co‐opted into novel functions that might have led to evolutionary novelties, at least in gastropods and cephalopods.

## INTRODUCTION

Homeotic genes constitute key developmental regulators in the ontogenetic establishment of animal body plans. These genes, such as the Hox and ParaHox genes, contain a homeobox or homeodomain coding sequence that encodes transcription factors, which specify and determine, via various downstream genes, the identity of body regions along the anteroposterior axis (e.g., Scott et al., 1989; McGinnis and Krumlauf, 1992; Gehring et al., 1994; Carroll, 1995; Brooke et al., 1998; Ferrier and Holland, 2001; Garcia‐Fernàndez, 2005; Hueber and Lohmann, 2008; Choo and Russell, 2011).

In bilaterians, studies on the spatial and temporal expression pattern of Hox and ParaHox genes are mainly available for two of the three major clades, namely Deuterostomia (e.g., Prince et al., 1998; Lowe et al., 2003; Garcia‐Fernàndez, 2005; Aronowicz and Lowe, 2006) and Ecdysozoa (e.g., Wang et al., 1993; Averof and Akam, 1995; Averof and Patel, 1997; Orii et al., 1999; Peterson et al., 1999). Detailed Hox and ParaHox gene expression data within the third bilaterian superclade, Lophotrochozoa, are available to a much lesser degree such as for various annelid and two nemertean species (Nardelli‐Haefliger and Shankland, 1992; Nardelli‐Haefliger et al., 1994; Wong et al., 1995; Kourakis et al., 1997; Irvine and Martindale, 2000; Kulakova et al., 2007; Fröbius et al., 2008; Bakalenko et al., 2013; Gharbaran et al., 2013; Hiebert and Maslakova, 2015a,b).

As for the majority of lophotrochozoan phyla, however, in Mollusca, the phylum with the widest spectrum of body plans, research into Hox and ParaHox gene expression is still in its infancy (Biscotti et al., 2014; Wanninger and Wollesen, 2015). A maximum of 11 Hox and three ParaHox genes were identified in gastropods (Giusti et al., 2000; Barucca et al., 2003, 2006; Hinman et al., 2003; Canapa et al., 2005; Pérez‐Parallé et al., 2005; Iijima et al., 2006; Pernice et al., 2006; Biscotti et al., 2007; Samadi and Steiner, 2010a,b; Simakov et al., 2013) and, most recently, in one bivalve (Takeuchi et al., 2016), 11 Hox and two or three ParaHox genes in other bivalves (Barucca et al., 2003, 2006; Canapa et al., 2005; Pérez‐Parallé et al., 2005; Iijima et al., 2006; Pernice et al., 2006; Biscotti et al., 2007; Zhang et al., 2012; De Oliveira et al., in review), nine Hox and two ParaHox genes in scaphopods (Iijima et al., 2006; Wollesen et al., 2015a), and ten Hox and three ParaHox genes in cephalopods (Callaerts et al., 2002; Lee et al., 2003; Iijima et al., 2006; Pernice et al., 2006; Biscotti et al., 2007; Wollesen et al., 2015a). In the octopod *Octopus bimaculoides*, eight Hox genes (the number of the ParaHox genes remains unknown) were identified (Albertin et al., 2015). Within the aculiferans, in Polyplacophora nine Hox and three ParaHox genes (Barucca et al., 2006; Iijima et al., 2006; Biscotti et al., 2007), in Solenogastres seven to eight Hox genes, and in Caudofoveata four Hox genes were identified, and at least one Parahox gene in the latter taxon (Iijima et al., 2006). However, detailed data on the tempospatial expression of these genes are known for very few species only (Giusti et al., 2000; Hinman et al., 2003; Lee et al., 2003; Le Gouar et al., 2003; Samadi and Steiner, 2009, 2010a,b; Focareta et al., 2014; Fritsch et al., 2015).

In contrast to other bilaterians, the gastropod and cephalopod Hox and ParaHox gene expression data suggest that these genes have been co‐opted into the formation of distinct organs such as the mantle, shell, radula, or the light organ of certain squids (Giusti et al., 2000; Hinman et al., 2003; Lee et al., 2003; Le Gouar et al., 2003; Samadi and Steiner, 2009, 2010a,b; Focareta et al., 2014). Recent data on the polyplacophoran *Acanthochitona crinita* showed that the Hox genes have preserved their hypothetical ancestral mode of expression, which is in a colinear manner along the anteroposterior axis (Fritsch et al., 2015). Herein, we describe the expression of the three missing lophotrochozoan‐specific central class Hox genes *Lox5, Lox4*, and *Lox2*, together with the ParaHox gene *caudal* (*Cdx*), which is often believed to have a function in hindgut formation (Brooke et al., 1998; Holland, 2001; de Rosa et al., 2005; Kulakova et al., 2008; Hui et al., 2009), in the polyplacophoran *A. crinita*.

## MATERIALS AND METHODS

### Collection, Fixation, and Terminology

Adult individuals of *Acanthochitona crinita* were collected in the intertidal zone along the coastline of the Biological Station Roscoff in France. Spawning was induced by water temperature variations and sun light exposure. Eggs were fertilized with a concentrated sperm solution for 30 min and reared at 21–23°C. Animals were fixed in 4% paraformaldehyde in MOPS buffer, dehydrated by a graded methanol series, and stored in 100% methanol at −20°C (see Fritsch et al., 2015). For whole‐mount immunostaining, larvae were fixed in 4% paraformaldehyde for 45 min at room temperature, dehydrated, and stored in 100% methanol at 4°C.

The entirely lecithotrophic larval development was divided into three different larval stages. Early‐stage trochophore larvae are equipped with an apical tuft and a prototroch, which divides the larva into an anterior episphere and a posterior hyposphere. Mid‐stage trochophore larvae are slightly longer than the earliest stage, about 280 μm, and the anlagen of the ventral foot and the dorsal shell plates are discernible in the hyposphere region. Late‐stage trochophore larvae are approximately 360 μm in length and seven dorsal shell plate anlagen are present in the hyposphere. At the end of larval development, larvae undergo metamorphosis and commence their benthic lifestyle. Herein, terminology and descriptive larval terms are used following Fritsch et al. (2015).

### Orthology Assignment and Phylogenetic Analysis

**Figure 1:**
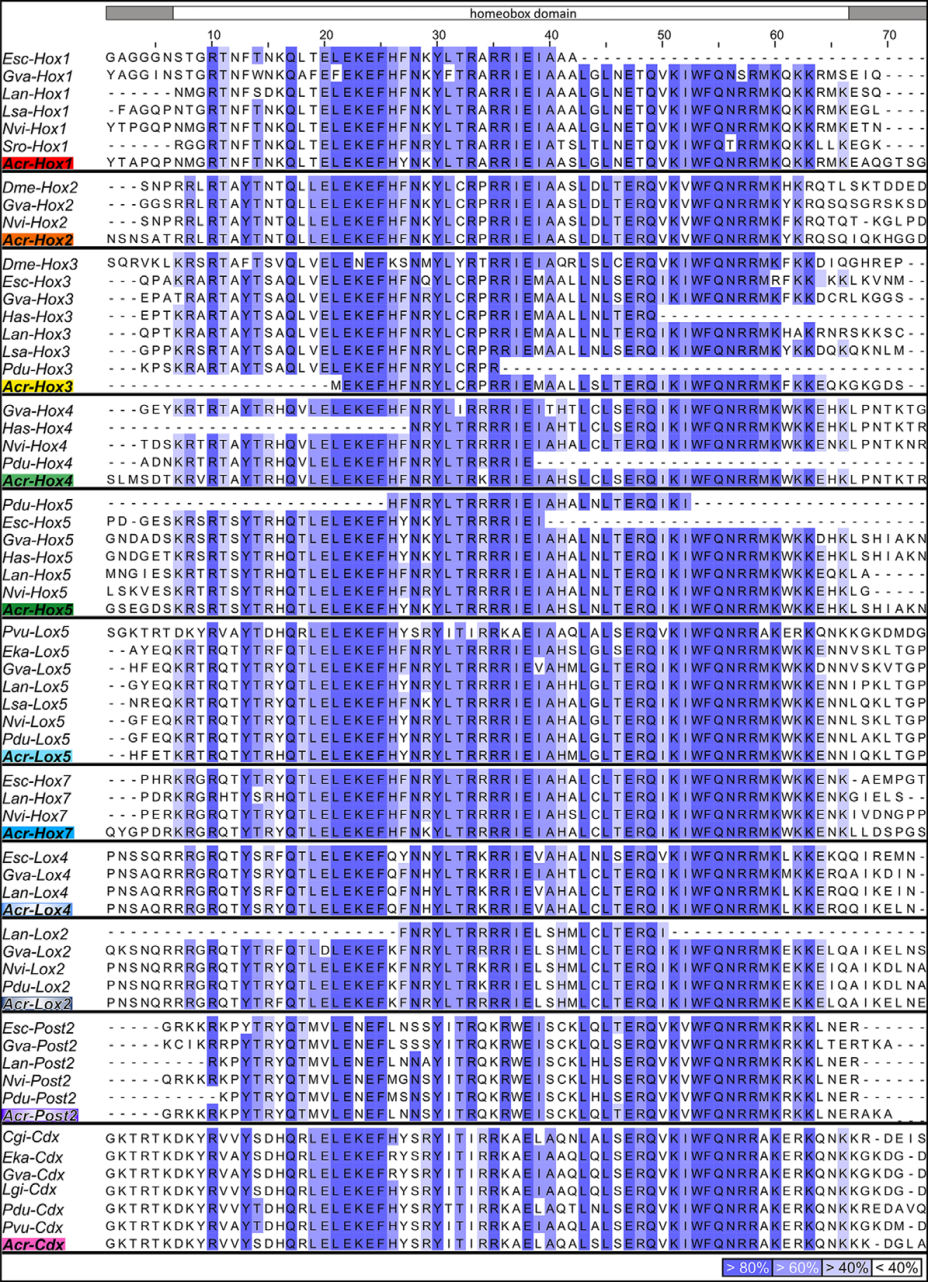
Homeodomain sequence alignment. All identified *Acanthochitona crinita* Hox and *Cdx* homeodomain sequences are aligned with their respective bilaterian orthologs. Residues are bluish colored in each column according to the percentage of identity that agrees with the consensus sequence. Residues with less than 40% of identity are not colored. Dashes represent missing data. *Acr*, *Acanthochitona crinita*; *Cgi*, *Crassostrea gigas*; *Dme*, *Drosophila melanogaster*; *Eka*, *Euperipatoides kanangrensis*; *Esc*, *Euprymna scolopes*; *Gva*, *Gibbula varia*; *Has*, *Haliotis asinina*; *Lan*, *Lingula anatina*; *Lsa*, *Lineus sanguineus*; *Nvi*, *Nereis virens*; *Pdu*, *Platynereis dumerilii*; *Pvu*, *Patella vulgata*; *Sro*, *Symsagittifera roscoffensis*.

**Figure 2:**
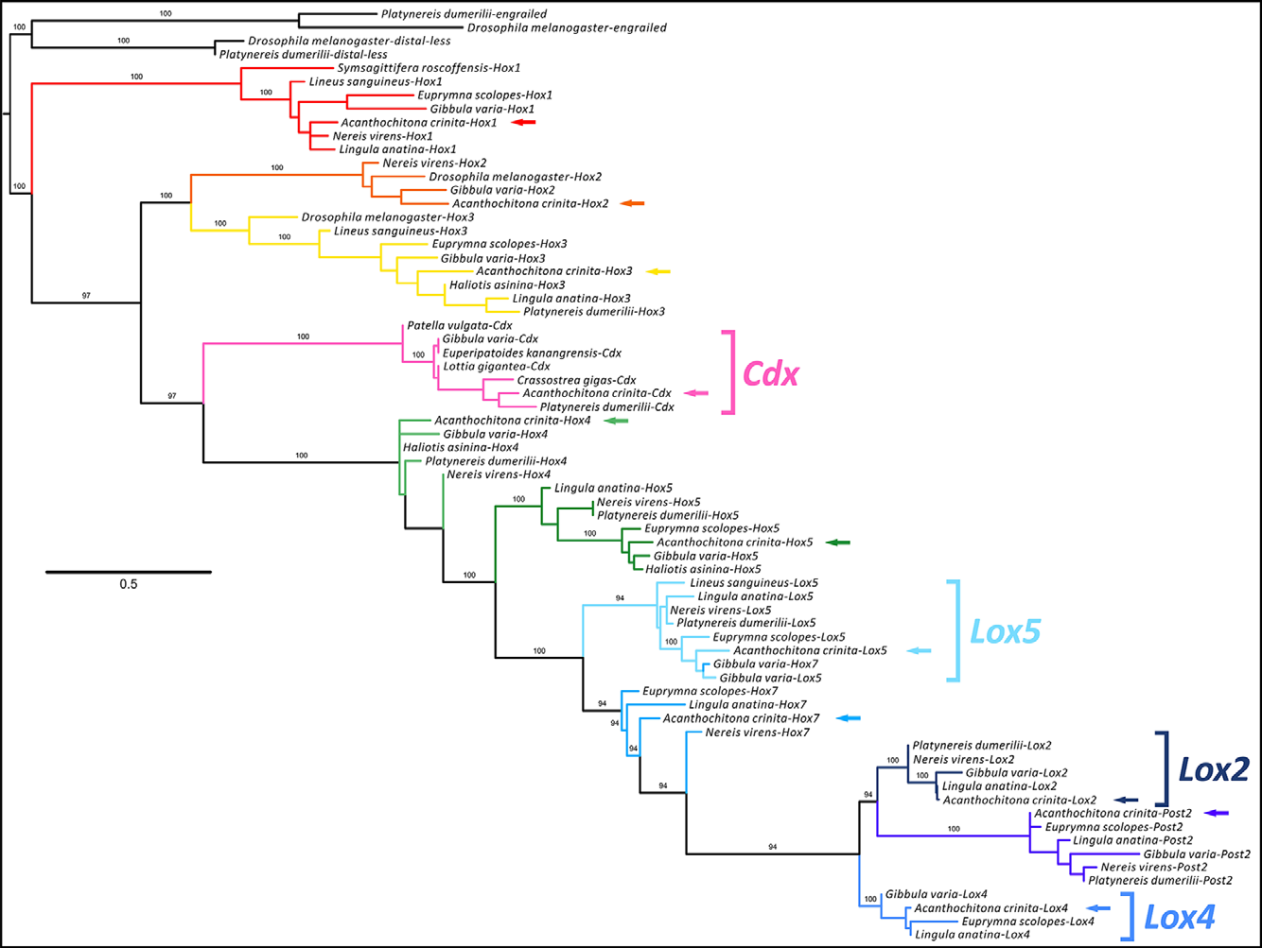
Phylogeny of homeodomain genes. Phylogeny of Hox genes and *Cdx* gene families from amino acid sequences present in the homeodomain. The best fit tree was inferred by a maximum likelihood phylogenetic analysis with the RAxML v7.2.6 software; bootstrap support values over 90% are displayed. All identified *Acanthochitona crinita* Hox and ParaHox genes within the respective gene clusters are highlighted by colored arrows. For the genes of interest of our study, the Lox and *Cdx* genes are highlighted by colored brackets. All *A. crinita* Hox and ParaHox gene sequences cluster with appropriate bilaterian Hox gene orthologs. The homeotic genes *distal‐less* and *engrailed* of *Platynereis dumerilii* and *Drosophila melanogaster* are used as outgroups.

Local similarity searches with amino acid sequences of other organisms retrieved from NCBI against a transcriptome of *A. crinita* (Trinity assembled) were performed using the program Geneious 6.1.6 (Biomatters Ltd., Auckland, New Zealand). The multiple amino acid sequence alignment of the herein identified Lox genes and the *Cdx* gene (NCBI accession numbers: *Acr‐Lox5*, KU960944; *Acr‐Lox4*, KU960945; *Acr‐Lox2*, KU960946; *Acr‐Cdx*, KU960947), the already identified Hox genes in *A. crinita* (see Fritsch et al., 2015), and their metazoan orthologs was performed with the program mafft v7.221 (Katoh et al., 2005), while Jalview 2 (Waterhouse et al., 2009) was used to illustrate the alignment (Fig. 1). For identification of the homeodomain sequences of *A. crinita*, a maximum likelihood analysis using a Jones–Taylor–Thornton (Jones et al., 1992) amino acid substitution model with 1,000 replicates was performed within the RAxML v7.2.6 software (Stamatakis, 2006) (Fig. 2).

### Molecular and Immunostaining Experiments

Specific Acr‐Lox gene and *Acr‐Cdx* primers were designed with Geneious 6.1.6. PCR amplifications, cloning and ligation were performed as described in Fritsch et al. (2015). Probes were designed with a DIG‐labeling kit (#11277073910, Roche Diagnostics GmbH, Mannheim, Germany). For whole‐mount in situ hybridizations, *A. crinita* larvae were decalcified, pretreated with proteinase‐K solution, and washed several times in phosphate buffer‐based solutions. For reduction of probe charge, larvae were incubated in a 1% triethanolamine and 0.5% acetic anhydride solution. After preincubation overnight in 100% hybridization buffer, larvae were hybridized with a probe concentration of 0.25 ng/μL at 60°C in a water bath for 48 hr. Subsequently, larvae were washed and rinsed with a descending SSC washing buffer, then several times in a maleic acid buffer‐based solution. The digoxigenin antibody conjugated to alkaline phosphatase incubation (#11093274910, Roche; 1:5,000 dilution) was carried out overnight at 4°C and for transcript visualization larvae were transferred into a color reaction buffer (7.5% polyvinyl alcohol with 2% NBT/BCIP (#11681451001, Roche)) for 45–60 min. Larvae were cleared in a 1:1 benzylalcohol:benzylbenzoate solution (for further details, see Fritsch et al., 2015).

For staining of neural components, larvae were pretreated in a 4% Triton‐X 100 in PBS solution. To label acetylated α‐tubulin structures, a monoclonal mouse primary antibody (#T6793, Sigma‐Aldrich, St. Louis, Missouri, USA; 1:250 dilutions in PBT for 48 hr) together with an Alexa568‐coupled mouse secondary antibody (#A11004, Invitrogen, Carlsbad, CA, USA; dilution 1:300 in PBT for 48 hr) was used. The neurotransmitter serotonin was labeled with a polyclonal rabbit primary antibody (#S5545, Sigma; 1:250 dilutions in PBT for 48 hr) together with an Alexa633‐coupled rabbit secondary antibody (#A21070, Invitrogen; dilution 1:300 in PBT for 48 hr). SYBR Green‐I Nucleic Acid Gel Stain was used as nuclear counterstain (#S‐7567, Thermo Fisher Scientific, Waltham, MA, USA; 1:600 dilutions in PBS for 60 min). Specimens were mounted on microscope slides in Vectashield mounting medium (Vector Labratories, Burlingame, CA, USA) and scanned with a Leica DMI6000 CFS microscope equipped with a Leica TCS SP5 II scanning system (Leica Microsystems, Wetzlar, Germany). Scans were edited with IMARIS 7.3.1 (Bitplane, Zurich, Switzerland) and figures were designed using Corel Graphic Suite X3 (Corel Corporation, Ottawa, Canada).

## RESULTS

### Hox and ParaHox Gene Expression in *A. crinita*

**Figure 3:**
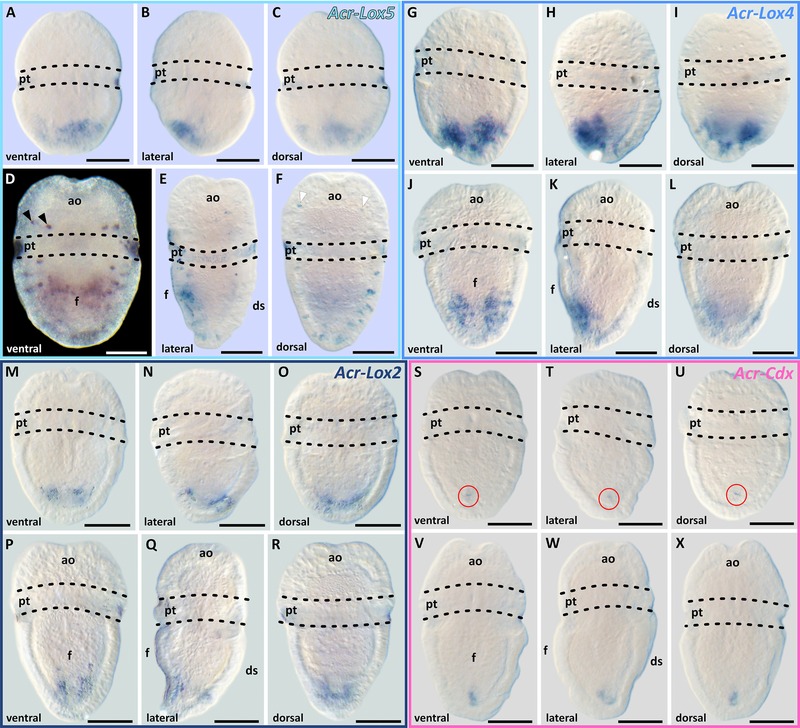
Acr‐Lox and *Acr‐Cdx* gene expression pattern in early‐ and mid‐stage trochophore larvae of *Acanthochitona crinita*. Apical faces up. The expression pattern of each gene is depicted in early‐ (upper row) and mid‐stage (bottom row) trochophore larvae of *A. crinita*, respectively (scale 50 μm). (A–F) *Acr‐Lox5* is expressed in epidermal and subepidermal cells mainly in the posterior part of the ventral hyposphere. In the ventral episphere, four *Acr‐Lox5*‐positive cells are present in the same area as the FMRF‐positive cells and the ampullary sensory system. Dorsally in the episphere (white arrows) and hyposphere single *Acr‐Lox5* transcript‐containing cells are present. (G–H) The expression pattern of *Acr‐Lox4* in the hyposphere appears farther posterior than the expression of *Acr‐Lox5*. In mid‐stage trochophore larvae, *Acr‐Lox4* transcripts are mainly distributed ventrally in epidermal and subepidermal cells of the posterior hyposphere region. (M–R) Small domains of *Acr‐Lox2* transcripts are present ventrally in subepidermal cells at the posterior end of the hyposphere, slightly posterior to that of *Acr‐Lox4*. (S–X) *Acr‐Cdx* is expressed in central subepidermal cells of the prospective developing hindgut at the posterior end of the hyposphere. ao, apical organ; ds, dorsal shell plates; f, foot; pt, prototroch.

The Hox genes *Acr‐Lox5*, *Lox4*, and *Lox2* and the ParaHox gene *Acr‐Cdx* are expressed in distinct domains of early‐stage *A. crinita* trochophore larvae. All three Hox genes are expressed in the posterior region of the ventral hyposphere (Figs. 3A–C, G–I, and M–O). The strongest expression pattern is that of *Acr‐Lox4*, which is present in two prominent epidermal and subepidermal cellular strands at the posterior pole of the larva (Figs. 3G–I). The expression pattern of *Acr‐Cdx* in early‐stage trochophore larvae is restricted to a subepidermal spot near the posterior pole of the larval body (Figs. 3S–U).

**Figure 4:**
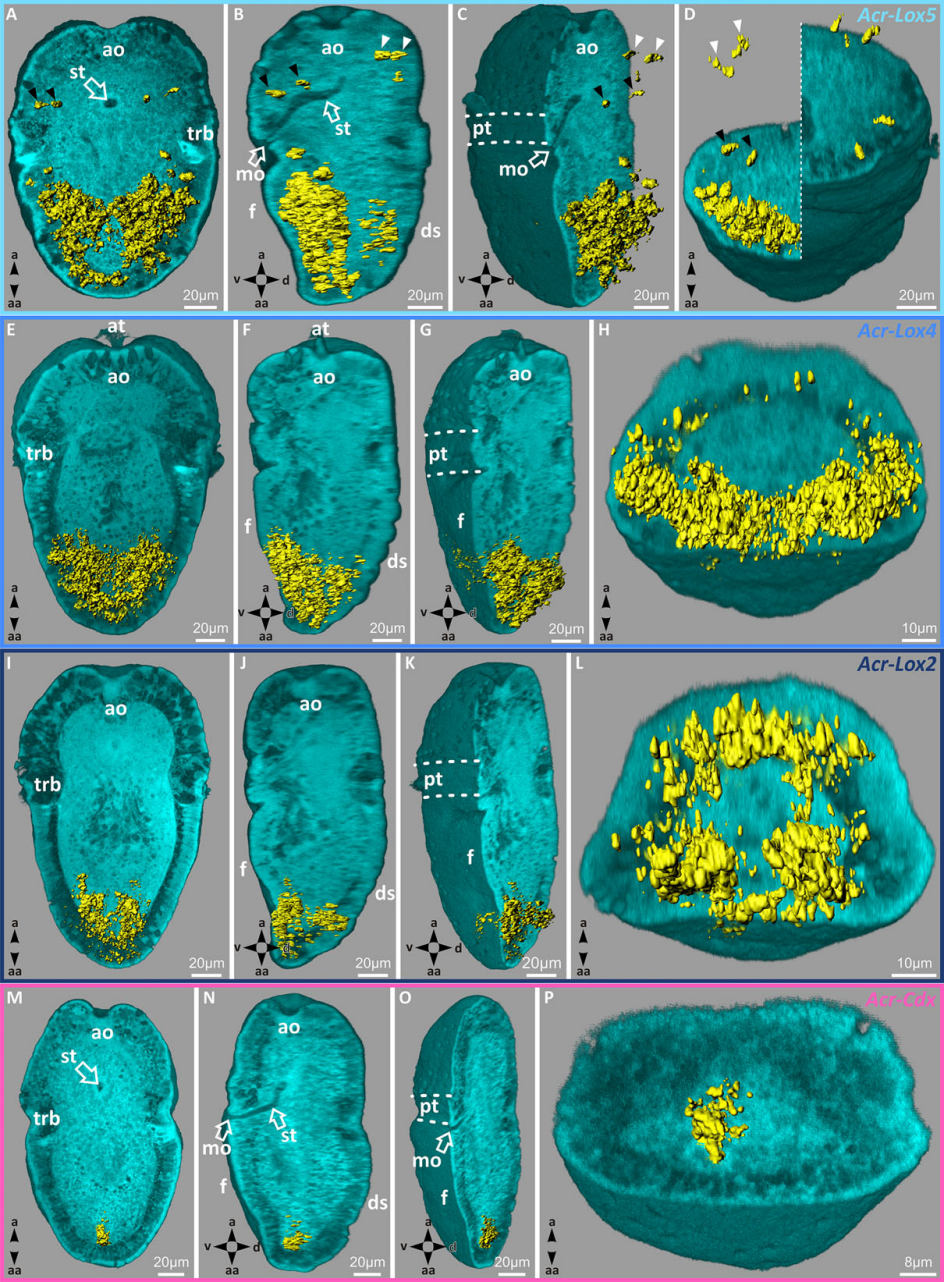
Acr‐Lox and *Acr‐Cdx* transcript distribution pattern in mid‐stage trochophore larvae of *Acanthochitona crinita*. Three‐dimensional reconstruction and localization of the specific gene expression pattern (yellow) within mid‐stage trochophore larvae of *A. crinita*. Morphology of the larvae is presented by autofluorescence images (cyan). From left to right, first column: longitudinal, second column: sagittal, third column: laterosagittal, and the forth column: transversal plane. (A–D) *Acr‐Lox5* transcription products present in ventral and dorsal subepidermal cell layers. In the ventral episphere, four single *Acr‐Lox5* transcript‐containing cells are present (white arrows) in the same area as *Acr‐FMRF* and ampullary sensory cells. In the dorsal episphere, also four single *Acr‐Lox5‐*positive cells (black arrows) are present. (E–H) *Acr‐Lox4* expressed within ventral and dorsal subepidermal cell layers in the posterior part of the hyposphere. (I–L) The expression pattern of *Acr‐Lox2* in ventral and dorsal subepidermal cell layers at the posterior end of the hyposphere. (S–X) *Acr‐Cdx* transcripts are present in subepidermal cells in the region of the prospective hindgut. a, apical; aa, ab‐apical; ao, apical organ; at, apical tuft; d, dorsal; ds, dorsal shell plates; f, foot; mo, mouth opening; pt, prototroch; st, stomodaeum; trb, trochoblast(s); v, ventral.

In mid‐stage trochophore larvae, transcripts of *Acr‐Lox5* are largely distributed in epidermal and subepidermal cell layers in the ventral hyposphere. Expression occurs in two prominent domains in the central and posterior region of the hyposphere (Figs. 3D–F and 4A–D). Transcripts are also present in individual ventrolateral cells, immediately posterior to the prototroch, and in several cells on the dorsal side of the hyposphere (Fig. 3F). In the episphere, four pairs of *Acr‐Lox5* transcript‐containing cells are present (Figs. 3D and F). Two pairs of ventrolateral cells and two pairs of dorsolateral cells are identifiable (Figs. 3D, F, and 4A–D, black and white arrowheads). In mid‐stage trochophore larvae, *Acr‐Lox4* is expressed in the posterior hyposphere in two parallel epidermal and subepidermal expression domains (Figs. 3J–L and 4E–H). Small *Acr‐Lox4* expression domains are present subepidermally in the dorsoposterior hyposphere (Figs. 4F and H). The expression pattern of *Acr‐Lox2* in the posterior hyposphere is less prominent than that of *Acr‐Lox4*. Two slender subepidermal cellular domains are present ventrally, and dorsally a faint *Acr‐Lox2* subepidermal expression domain is discernible (Fig. 3P–R and 4I–L). The expression pattern of *Acr‐Cdx* in mid‐stage trochophore larvae is still only detectable in a single subepidermal expression domain near the posterior pole of the larval body, most likely in the developing posterior digestive system (hindgut) (Figs. 3V–X and 4M–P).

**Figure 5:**
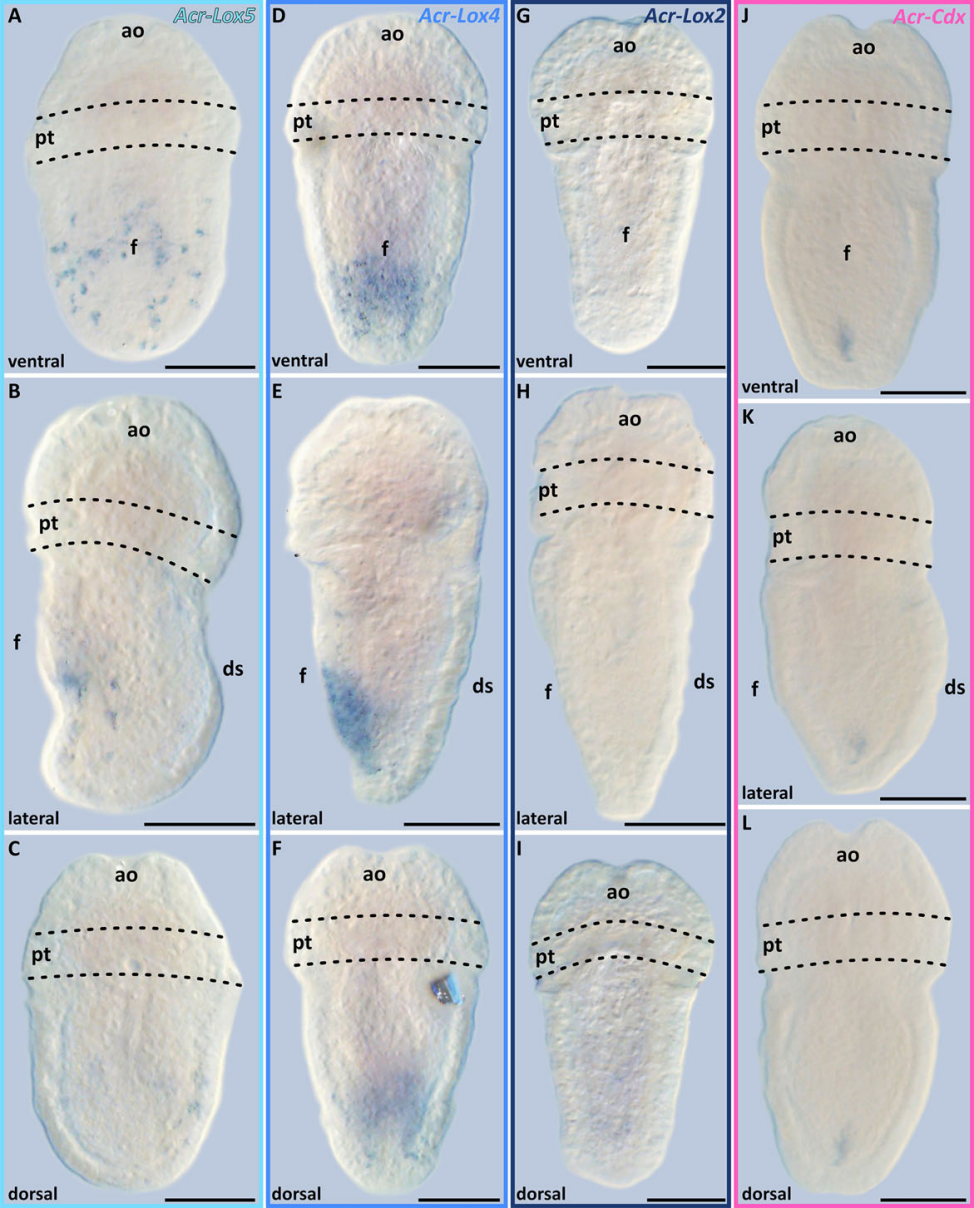
Acr‐Lox and *Acr‐Cdx* gene expression patterns in late trochophore larvae of *Acanthochitona crinita*. In late‐stage trochophore larvae, the expression level of all Acr‐Lox and the *Acr‐Cdx* gene gradually decreases (scale 50 μm). (A–C) *Acr‐Lox5* transcripts are only present in ventral epidermal cells within the ventral foot region. (D–F) *Acr‐Lox4* is weakly expressed in epidermal and subepidermal cells within the posterior ventral foot region. (G–I) *Acr‐Lox2* shows no expression signal in late‐stage trochophore larvae. (J–L) *Acr‐Cdx* is weakly expressed in subepidermal cells in the prospective hindgut at the posterior end of the larva. ao, apical organ; ds, dorsal shell plates; f, foot; pt, prototroch.

In late‐stage trochophore larvae, the expression levels of all four genes gradually decrease (Figs. 5A–L). In late trochophore larvae, *Acr‐Lox5* transcripts are only present in some ventral subepidermal cells within the foot region (Figs. 5A–C). A faint *Acr‐Lox4* expression is present in late‐stage trochophore larvae in ventral epidermal and subepidermal cells of the posterior foot region (Figs. 5D–F). No *Acr‐Lox2* expression was found in late‐stage trochophore larvae (Figs. 5G–I). In late‐stage trochophore larvae *Acr‐Cdx* is still, albeit weakly, expressed in subepidermal cells in the region of the prospective hindgut (Figs. 5J–L).

### *Elav* and *FMRF* Expression in the Developing Nervous System of *A. crinita*

**Figure 6:**
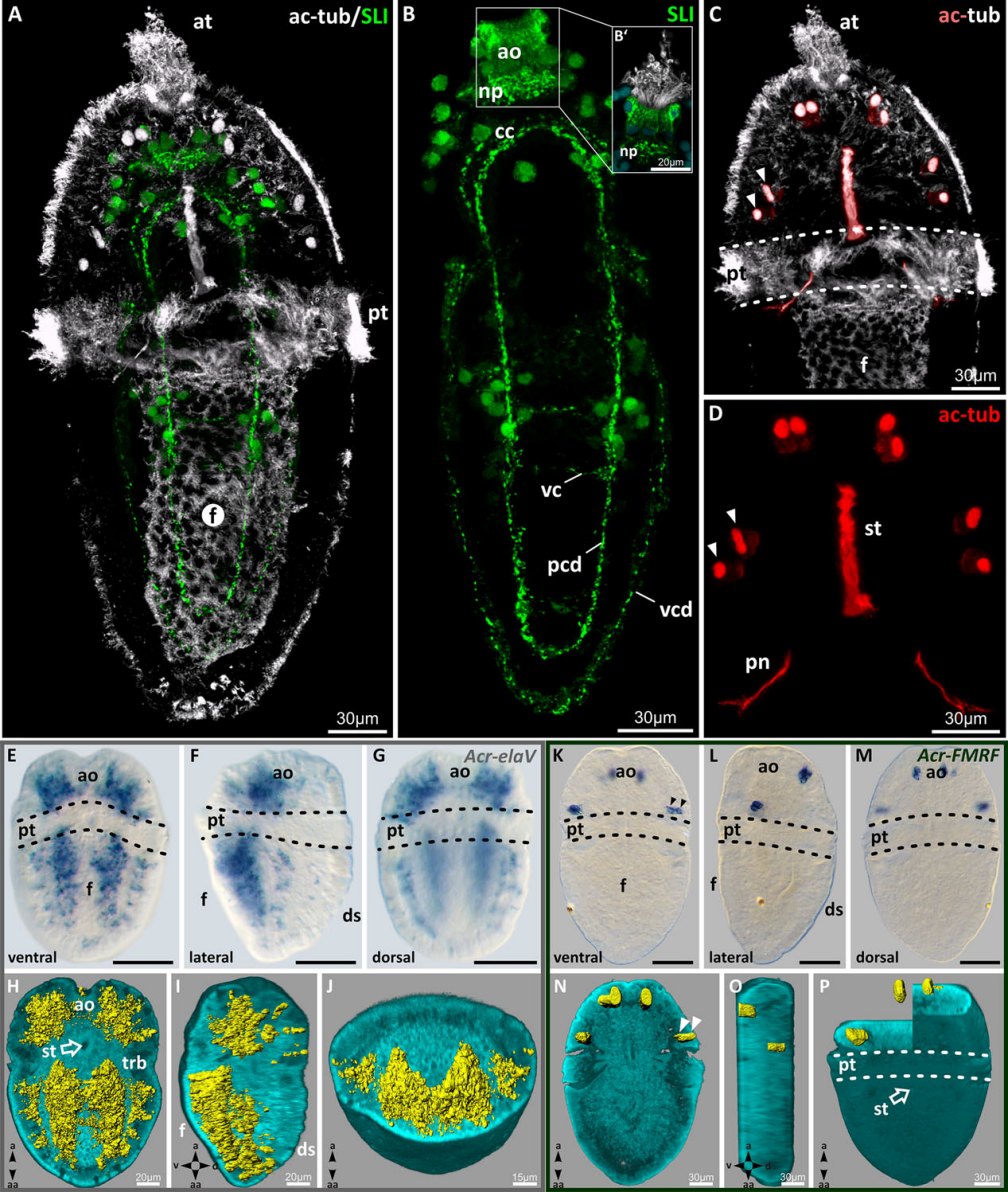
Nervous system staining and the *Acr‐Elav*/*Acr‐FMRF* expression patterns in mid‐stage trochophore larvae of *Acanthochitona crinita*. (A–D) Immunostaining of the nervous system (serotonin and acetylated α‐tubulin) in trochophore larvae. (E–J) Expression pattern of the *Acr‐Elav* in mid‐stage trochophore larvae. (K–P) Expression pattern of *Acr‐FMRF* in mid‐stage trochophore larvae. (H–J, N–O) From left to right, longitudinal, sagittal, and transversal plane. (A) Serotonin‐like immunoreactive (ir) labeled tetraneural nervous system (green) and tubulin‐containing cilia (white/red). (B and B′) Detailed serotonin‐positive tetraneural nervous system. Anteriorly, apical organ consisting of apical organ cells and neuropil. (C and D) Color‐coded reconstruction of the tubulin‐containing structures (red/white). Anterior cells of the ampullary sensory system, stomodaeum, and protonephridial canals. (E–G) Light micrograph of mid‐stage trochophore larvae showing *Acr‐Elav* expression pattern of the developing tetraneural nervous system (scale 50 μm). (H–J) Autofluorescence (cyan) of mid‐stage trochophore larvae and specific *Acr‐Elav* transcription product distribution (yellow) within the larval body. (K–M) Light micrograph of mid‐trochophore larvae showing the expression of *Acr‐FMRF* in the cells (arrows) of the ampullary system (scale 50 μm). (N–P) Autofluorescence (cyan) of mid‐stage trochophore larvae and specific *Acr‐FMRF* transcription product distribution (yellow) within the episphere of the larval body. a, apical; aa, ab‐apical; ao, apical organ; at, apical tuft; ac‐tub, acetylated α‐tubulin; cc, cerebral commissure; d, dorsal; ds, dorsal shell plates; f, foot; np, neuropil; pcd, pleurovisceral nerve cord; pn, protonephridia; pt, prototroch; st, stomodaeum; SLI, serotonin‐like immunoreactivity; trb, trochoblast(s); v, ventral; vc, ventral commissure; vcd, visceral nerve cord.

In trochophore larvae of *A. crinita*, a developing tetraneural nervous system is present (Figs. 6A–D). Immunostaining against 5HT (serotonin) revealed an apical organ (consisting of most probably monopolar neurons and a neuropil) and the anlage of the cerebral commissure at the anterior pole of the larva (Fig. 6B). Posterior to the commissure, four longitudinal neurite bundles (two ventromedial pedal and two ventrolateral visceral nerve cords) interconnected by transversal commissures are present (Figs. 6A and B). In addition to that, by using antibodies against α‐acetylated tubulin, the tubulin‐containing cells of the polyplacophoran‐specific larval ampullary sensory system (Haszprunar et al., 2002) was labeled in the anterior region of the episphere (Figs. 6C and D). Altogether, four ventrolateral and four dorsomedial tubulin‐containing cells are present.

The *Elav* expression pattern in trochophore larvae of *A. crinita* appears to colocalize with the immunostaining of the tetraneural nervous system (Figs. 6E–J). Ventrally in the episphere, transcripts of *Acr‐Elav* are present in two distinct domains, posterolaterally to the apical organ (Figs. 6E and F). The prototroch region is devoid of *Acr‐Elav* expression (Fig. 6E). In the ventral hyposphere, two prominent putative neuroectodermal medial longitudinal and two slender, more laterally positioned longitudinal expression strands are present (Figs. 6E and H).

Transcripts of the *FMRF* gene in larvae of *A. crinita* are mainly present in the epidermal cell layers of the episphere (Figs. 6K–P). Two pairs of ventrolateral (Figs. 6K and N) and two pairs of dorsomedial cells (Figs. 6M and P) contain *Acr‐FMRF* transcripts. These *Acr‐FMRF*‐containing cells appear to colocalize with the cells of the ampullary sensory system.

## DISCUSSION

### Identification of Hox and ParaHox Genes in *A. crinita*

The identification of a maximum of 11 Hox genes and three ParaHox genes in mollusks and annelids as their potential sister group might suggest that this was the situation in the last common ancestor of mollusks (Biscotti et al., 2014; Wanninger and Wollesen, 2015; Takeuchi et al., 2016). However, in the transcriptome of *A. crinita*, only ten Hox genes and one ParaHox gene were identified (present study; Fritsch et al., 2015; De Oliveira et al., in review). Although the gene *Post1* was identified in almost all molluscan class‐level lineages (Iijima et al., 2006), in *A. crinita* it was not found. Comprehensive BLAST searches were also performed in order to identify the orthologous sequences of the ParaHox genes *Gsx* and *Xlox*; however, these two genes were not recovered from our transcriptomic dataset. Additional BLAST and annotation investigations were performed with the program BUSCO (v1.1). BUSCO enables similarity searches between a transcriptome and a set of orthologous genes conserved in the Metazoa (Simão et al., 2015). The results showed that in the transcriptome of *A. crinita* about 95% conserved orthologous genes were identified, indicating that the transcriptome has a great depth and is almost complete. The definite presence or absence of *Post1* and the two Parahox genes in *A. crinita* may only—if at all—be assessed once the genome of the species becomes available.

### Hox gene expression in putative (neuro)‐ectodermal domains of polyplacophorans

Hox and ParaHox genes are key determinants for the formation of the anteroposterior body axis in the vast majority of bilaterian animals (e.g., Holland, 2001; Hughes and Kaufman, 2002; Garcia‐Fernàndez, 2005; Fröbius and Seaver, 2006; Aronowicz and Lowe, 2006; Kulakova et al., 2007, 2008; Hui et al., 2009; Fritsch et al., 2015). Expression studies show that these homeotic genes are also mainly expressed in the forming cells of the ectoderm, in particular the neuroectoderm (e.g., Kourakis et al., 1997; Hinman et al., 2003; Lee et al., 2003; Lowe et al., 2003; Kulakova et al., 2007, 2008; Samadi and Steiner, 2009, 2010a,b; Bakalenko et al., 2013). To localize Acr‐Lox gene transcripts in ectodermal and neuroectodermal derivatives of *A. crinita*, the formation of the nervous system was also documented by immunostaining techniques and by analyzing the gene expression patterns of *Elav* and *FMRF*. The Elav protein is first detectable in young neurons and studies in the fruit fly *Drosophila* revealed that Elav is not detected in other tissue types (e.g., Robinow and White, 1991; Berger et al., 2007).

The expression domains of the genes *Acr‐Lox5*, *Lox4*, and *Lox2* in the ventroposterior hyposphere overlap partly with the tetraneural nervous system. Within the area of the posterior developing pedal nerve cords, also the three Acr‐Lox genes are expressed. In addition, in the episphere of *A. crinita* larvae, the *Acr‐Lox5‐*containing ventral and dorsal cells most probably colocalize with the ventral and dorsal cells of the ampullary sensory system. Thus, together with the homeotic gene *Pax2/5/8*, *Acr‐Lox5* also seems to play a role in the formation of the ampullary sensory system (present study; Wollesen et al., 2015b).

Transcripts of *Acr‐Elav* in larvae of *A. crinita* are present within the area of the forming tetraneural nervous system. In particular, in the hyposphere, all four longitudinal nerve cords overlap with the *Acr‐Elav* expression pattern. Furthermore, transcripts of *Acr‐Elav* in the posterior hyposphere region also colocalize with the medial longitudinal expression domains of the genes *Acr‐Lox5*, *Lox4*, and *Lox2*.

Transcripts of *Acr‐FMRF* in larvae of *A. crinita* are present within the ventral and dorsal ampullary sensory cells in the episphere. The matching expression pattern of *Acr‐FMRF* and *Acr‐Lox5* further substantiates the assumption that the transcripts of this gene are also localized within the cells of that particular sensory structure.

Altogether, the expression patterns of the genes *Acr‐Lox5*, *Lox4*, and *Lox2* overlap and colocalize partly with the developing nervous system and with the expression patterns of *Acr‐Elav* and *Acr‐FMRF*. Thus, the herein investigated Acr‐Lox genes are primarily expressed in ectodermal and neuroectodermal domains, a condition which is similar to the other Hox genes in *A. crinita* (see Fritsch et al., 2015). Nevertheless, as already mentioned for the formerly studied Hox genes in *A. crinita*, the presence of Acr‐Lox gene transcription products in endo‐ and mesodermal cell layers cannot be excluded. Next to the tissue of the developing nervous system, transcripts from all three Lox genes are also present within the ventral region of developing muscle fibers and within the central area of the forming digestive tract (see also Fritsch et al., 2015).

### Comparison of Lox Gene Expression within Mollusca

To date, Lox gene expression studies in mollusks are only available for the gastropod *Gibbula varia* and the cephalopod *Euprymna scolopes* (Lee et al., 2003; Samadi and Steiner, 2010a). Similar to the gastropod *G. varia*, the first transcription products of *Acr‐Lox5*, *Lox4*, and *Lox2* were found in early‐stage trochophore larvae of *A. crinita* immediately after hatching. In early‐ and mid‐stage trochophore larvae of *A. crinita*, all three Lox genes are expressed predominantly in the ventral ectoderm of the posterior hyposphere. Only *Acr‐Lox5* is additionally expressed in the episphere, namely in paired ventral and dorsal ectodermal cells. In contrast, in trochophores of *G. varia, Gva‐Lox5*, *Lox4*, and *Lox2* are expressed either in ectodermal cells in the episphere (*Gva‐Lox5* and *Lox2*), in the apical organ, and later in the forming cerebral ganglion (*Gva‐Lox5*, *Lox4*, and *Lox2*), or in the ciliated cells of the prototroch and later within the velum (*Gva‐Lox4* and *Lox2*) (Samadi and Steiner, 2010a).

Overall, the expression patterns of all three Lox genes differ significantly between gastropods and polyplacophorans with only *Lox5* showing a congruent expression pattern. In both *G. varia* and *A. crinita*, *Lox5* is expressed in the episphere. Nevertheless, in *A. crinita*, *Lox5* transcripts are present in four ventral and four dorsal cells, whereas *Gva‐Lox5* is expressed in two ventral and two dorsal cells at the base of the apical organ (Samadi and Steiner, 2010a).

In late‐stage trochophore larvae of *A. crinita*, the expression patterns of *Acr‐Lox5* and *Lox4* are rather faint and restricted to ventral ectodermal cells in the hyposphere. Transcripts of *Acr‐Lox2* seem to be entirely absent. In contrast, in pre‐ and posttorsional veliger stages of *G. varia*, all three Lox genes are prominently expressed in the cerebral ganglion and velum (Samadi and Steiner, 2010a). In the cephalopod *E. scolopes*, the gene *Esc‐Lox4* is expressed in parts of the central nervous system, within the pedal ganglion, and *Esc‐Lox5* shows an expression pattern in the brachial crown (Lee et al., 2003). The expression pattern of *Lox2* during cephalopod development is still unknown (Lee et al., 2003; Wanninger and Wollesen, 2015 for review).

**Figure 7:**
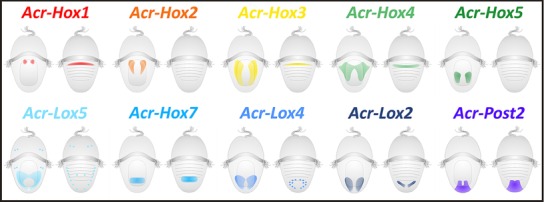
Schematic expression pattern summary of the ten Hox genes identified in *Acanthochitona crinita*. Hox gene transcription products in mid‐stage trochophore larvae appear in an anteroposterior gradient. Anterior Hox genes are expressed in the anterior part of the hyposphere, central‐class Hox genes in the middle part of the hyposphere, and posterior Hox genes at the posterior end of the hyposphere. Left image of each pair in ventral view and right image of each pair in dorsal view.

Altogether, the Acr‐Lox gene expression pattern in Polyplacophora compared with that in the gastropods and cephalopods indicates that the central Hox genes *Lox5*, *Lox4*, and *Lox2* in *A. crinita* seem to be primarily expressed in the (neuro‐)ectodermal cells or cell layers that contribute to the formation of neural tissues, but not exclusively in distinct structures of the nervous system, such as the apical organ or the cerebral commissure. Instead, in the polyplacophoran *A. crinita*, Lox genes are expressed in an anteroposterior colinear manner (Fig. 7). In accordance with the other polyplacophoran Hox genes, the Acr‐Lox genes are also expressed in defined body regions along the anteroposterior axis (Fritsch et al., 2015). This is in contrast to the condition found in conchiferan mollusks but resembles the condition found in other bilaterians (Lewis, ’78; Scott et al., 1989; McGinnis and Krumlauf, 1992; Wang et al., 1993; Carroll, 1995; Prince et al., 1998; Orii et al., 1999; Ferrier and Holland, 2001; Hughes and Kaufman, 2002; Lowe et al., 2003; Wray et al., 2003; Garcia‐Fernàndez, 2005). The opposing gastropod and cephalopod Lox gene expression patterns within distinct nervous system structures (e.g., apical organ, cerebral ganglia) or in locomotion tissues/structures (e.g., trochoblasts of the prototroch, brachial crown) indicate co‐option and functional plasticity of these genes at least in both conchiferan representatives.

Interestingly, throughout larval development of *A. crinita*, *Acr‐Lox5* is the only Hox gene that is expressed within the region of the episphere, however, not in cells of the apical organ. The entire lack of Acr‐Hox gene transcripts in the apical organ in Polyplacophora (see Fritsch et al., 2015 and herein) contradicts the hypothesis of Marlow and colleagues (2014) that Hox genes generally play a role in the formation of the apical organ in planktonic ciliated larvae, at least for this molluscan clade.

### Comparative Analysis of Hox Gene Expression in Polyplacophora and Other Lophotrochozoans

As in the polyplacophoran *A. crinita*, the Lox genes in polychaete annelids show a strict colinear anteroposterior expression pattern in the hyposphere of early trochophore larvae. In early trochophores of *Nereis virens* (now renamed as *Alitta virens*), the *Lox5* transcript is present in a similar ventral and posterior region of the hyposphere (Kulakova et al., 2007). *Nvi‐Lox4* and *Nvi‐Lox2* are first expressed in the pygidial area of *Nereis* (*Alitta*) nectochaete larvae and these genes do not seem to be involved in the formation of the presegmented larval body. Thereby, *Nvi‐Lox4* and *Nvi‐Lox2* are also expressed in a colinear manner (Kulakova et al., 2007). A colinear anteroposterior Lox gene expression pattern is also observed in other annelids, namely the sedentary *Capitella teleta* and the hirudinean *Hirudo medicinalis* and *Helobdella triserialis* (Nardelli‐Haefliger and Shankland, 1992; Kourakis et al., 1997; Gharbaran et al., 2013).

Together with the remaining Hox genes (see Fritsch et al., 2015), the Lox gene transcripts appear predominantly during the patterning processes in early‐ and mid‐stage trochophore larvae in *A. crinita*. As the other Hox genes in *A. crinita*, also the Acr‐Lox genes do not appear to be restricted to the ectodermal expression domains. Instead, they also seem to be present in developing endo‐ and mesodermal tissues (see also Fritsch et al., 2015). A similar expression domain of Lox genes in all three germ layers is present in early larval stages, prior to the onset of segmentation, of the polychaetes *Chaetopterus* sp., *C. teleta*, *Nereis* (*Alitta*) *virens*, and *Platynereis dumerilii*. Thereby, the transcripts of the remaining Hox genes are also present in endo‐ and mesodermal cell layers in early trochophore larvae (Irvine and Martindale, 2000; Kulakova et al., 2007; Fröbius et al., 2008). Correspondingly, in early and presegmental embryonic stages of *H. medicinalis*, *Helobdella robusta*, and *H. triserialis*, Hox gene expression appears in all three germ layers (Nardelli‐Haefliger and Shankland, 1992; Kourakis et al., 1997). Later, during segment formation processes in meta‐trochophore larvae of *Chaetopterus* sp., *C. teleta*, *Nereis* (*Alitta*) *virens*, and *P. dumerilii*, and in late embryonic stages of *H. medicinalis*, *H. robusta*, and *H. triserialis*, Hox gene expression appears particularly in the germ layers of newly differentiating segments in an anteroposterior gradient (Nardelli‐Haefliger and Shankland, 1992; Nardelli‐Haefliger et al., 1994; Wong et al., 1995; Kourakis et al., 1997; Irvine and Martindale, 2000; Kulakova et al., 2007; Fröbius et al., 2008; Bakalenko et al., 2013; Gharbaran et al., 2013). In contrast, in late‐stage trochophores of *A. crinita*, all identified Hox genes (including Lox genes) are only weakly expressed or expression is entirely lacking. During this stage, the anlagen of all major serially arranged muscular and neural features are established and are subsequently further elaborated during postmetamorphic development (Friedrich et al., 2002; Voronezhskaya et al., 2002; Wanninger and Haszprunar, 2002; Scherholz et al., 2013).

Apart from the annelids, the only other detailed and comprehensive lophotrochozoan Hox gene expression data are available for the nemertean species *Micrura alaskensis* and *Pantinonemertes californiensis*. Although in the pilidiophoran species *M. alaskensis* a clear anteroposterior Hox gene expression gradient is present only in the developing juvenile stages (but not during larval development), in the hoplonemertean species *P. californiensis* the genes *Hox1‐Hox4*, *Lox5*, and *Post2* are clearly expressed in larval and juvenile stages in a manner that suggests colinearity (Hiebert and Maslakova, 2015a,b). The pilidiophoran larva is considered an evolutionary novelty that may be patterned by genetic mechanisms other than the Hox genes (Hiebert and Maslakova, 2015a). Lox genes are similarly expressed in Nemertea, Polyplacophora, and Annelida, that is, near the posterior end of the larval or juvenile body (Hiebert and Maslakova, 2015a,b).

The comparison of the Hox and Lox gene data of *A. crinita* with data on the well‐investigated Annelida and Nemertea clearly revealed that Hox genes are expressed in a similar anteroposterior pattern during their early larval development (Nardelli‐Haefliger and Shankland, 1992; Nardelli‐Haefliger et al., 1994; Wong et al., 1995; Kourakis et al., 1997; Irvine and Martindale, 2000; Kulakova et al., 2007; Fröbius et al., 2008; Bakalenko et al., 2013; Gharbaran et al., 2013; Hiebert and Maslakova, 2015a,b). This anteroposterior expression pattern is in stark contrast to the condition found in Gastropoda and Cephalopoda.

### Comparative Aspects of *Cdx* Expression in Lophotrochozoa

The ParaHox gene *Cdx* (*caudal*) is often thought to pattern the posterior region of the digestive tract in bilaterian animals (Brooke et al., 1998; Holland, 2001; de Rosa et al., 2005; Fröbius and Seaver, 2006; Kulakova et al., 2008; Hui et al., 2009; Samadi and Steiner, 2010b; Altenburger et al., 2011). In addition, ParaHox gene expression studies in the gastropod *G. varia* and the annelids *C. teleta*, *Nereis* (*Alitta*) *virens*, and *P. dumerilii* showed that *Cdx*, as well as the two other representatives of the lophotrochozoan ParaHox gene cluster, *Gsx* and *Xlox*, is also associated with the development of the nervous system (de Rosa et al., 2005; Fröbius and Seaver, 2006; Kulakova et al., 2008; Hui et al., 2009; Samadi and Steiner, 2010b).

In *A. crinita, Cdx* is expressed in all trochophore stages in the posteromedian hyposphere that probably represents an ectodermal domain and forms the prospective hindgut. This expression pattern is very similar to that of the trochophores of the gastropod *G. varia* and *Patella vulgata*, the polychaete annelids *Nereis* (*Alitta*) *virens* and *P. dumerilii*, the hoplonemertean *P. californiensis*, as well as the brachiopod *Terebratalia transversa* (Le Gouar et al., 2003; Kulakova et al., 2008; Hui et al., 2009; Samadi and Steiner, 2010b; Altenburger et al., 2011; Hiebert and Maslakova, 2015b). In *G. varia* and *P. vulgata*, *Gva‐Cdx* and *Pva‐Cdx* are also expressed in the cells of the neuroectoderm and the mesoderm (Le Gouar et al., 2003; Samadi and Steiner, 2010b). In *P. dumerilii*, *Pdu‐Cdx* is also expressed in mesodermal and potentially also in endodermal precursors (Hui et al., 2009). In *A. crinita*, such an expression is absent; however, the posterior *Cdx* expression in the trochophores of *A. crinita* matches the spatial expression pattern in most other protostomes (Brooke et al., 1998; Ferrier and Holland, 2001; de Rosa et al., 2005; Kulakova et al., 2008; Hui et al., 2009; Samadi and Steiner, 2010b; Altenburger et al., 2011; Hiebert and Maslakova, 2015b). Thus, the ParaHox gene *Cdx* seems to be involved in the formation of the posterior digestive system in the polyplacophoran *A. crinita*.

## CONCLUSIONS

As previously shown for the Hox genes *Acr‐Hox1‐5*, *Acr‐Hox7*, and *Acr‐Post2*, the Acr‐Lox genes are likewise expressed in a distinct anteroposterior manner in the polyplacophoran mollusk *A. crinita*, similar to the expression pattern in annelids and other bilaterians. This pattern differs from the expression in Gastropoda and Cephalopoda. These findings suggest that the Hox genes are involved in anteroposterior body axis patterning in Polyplacophora, similar to the proposed ancestral role of bilaterian Hox genes. The co‐option of Hox genes into the formation of specific morphological features seems to be a characteristic of Conchifera, at least of gastropods and cephalopods. Recent genomic data from the octopod *O. bimaculoides* (Albertin et al., 2015) have shown that Hox genes in this species are not arranged in a single cluster, which is in line with the nonanterior–posterior Hox gene expression pattern in gastropods and cephalopods. Whether this phenomenon of noncolinearity combined with distinct structural Hox gene expression domains was already present in the last common ancestor of Conchifera or is restricted to gastropods and cephalopods remains open until data on the scaphopods and bivalves become available. The *Cdx* expression pattern in the region of the forming hindgut of *A. crinita* is strikingly similar to that in chordates, ecdysozoans, and other lophotrochozoans and suggests an evolutionarily conserved function of *Cdx* in posterior digestive tract formation in bilaterian animals.
